# Supplementary material for: The Effect of a Planetary Health Diet on the Human Gut Microbiome: A Descriptive Analysis
Source: Nutrients. 2023 Apr 16;15(8):1924. doi: 10.3390/nu15081924 (PMC10144214; doi:10.3390/nu15081924)
Supplement: Supplementary file 1 [file nutrients-15-01924-s001.zip › nutrients-2310393-supplementary.docx]

| Supplementary Table S1: List of bacterial species identified by culturing and MALDI-TOF. Listed are all bacterial species that were cultured from native stool samples and identified via MALDI-TOF. The table is divided in the three dietary groups. |  |  |  |
| --- | --- | --- | --- |
| **Species** | **OV** | **PH** | **VV** |
| *Acinetobacter johnsonii* | 0 | 0 | 5 |
| *Anaerococcus vaginalis* | 0 | 0 | 1 |
| *Bacillus altitudinis* | 0 | 1 | 0 |
| *Bacillus amyloliquefaciens* | 0 | 1 | 0 |
| *Bacillus cereus* | 1 | 5 | 2 |
| *Bacillus licheniformis* | 1 | 0 | 0 |
| *Bacillus mojavensis* | 0 | 1 | 1 |
| *Bacillus pumilus* | 0 | 2 | 0 |
| *Bacillus subtilis* | 1 | 0 | 0 |
| *Bacillus velezensis* | 1 | 1 | 1 |
| *Bacteroides caccae* | 1 | 1 | 0 |
| *Bacteroides ovatus* | 0 | 1 | 0 |
| *Bacteroides thetaiotaomicron* | 0 | 3 | 0 |
| *Bacteroides uniformis* | 0 | 1 | 0 |
| *Bifidobacterium adolescentis* | 0 | 1 | 0 |
| *Bifidobacterium longum* | 2 | 3 | 2 |
| *Bifidobacterium pseudocatenulatum* | 1 | 0 | 0 |
| *Clostridium perfringens* | 3 | 3 | 3 |
| *Clostridium tertium* | 0 | 0 | 1 |
| *Collinsella aerofaciens* | 0 | 3 | 0 |
| *Corynebacterium glucuronolyticum* | 0 | 0 | 1 |
| *Eggerthella lenta* | 1 | 0 | 0 |
| *Enterobacter bugandensis* | 0 | 0 | 1 |
| *Enterobacter cloacae* | 0 | 2 | 0 |
| *Enterococcus avium* | 1 | 0 | 0 |
| *Enterococcus casseliflavus* | 0 | 0 | 2 |
| *Enterococcus faecalis* | 1 | 0 | 0 |
| *Enterococcus faecium* | 2 | 2 | 7 |
| *Enterococcus hirae* | 0 | 0 | 1 |
| *Enterococcus mundtii* | 0 | 0 | 2 |
| *Escherichia coli* | 9 | 26 | 8 |
| *Finegoldia magna* | 0 | 1 | 0 |
| *Hafnia alvei* | 0 | 1 | 0 |
| *Klebsiella pneumoniae* | 0 | 0 | 1 |
| *Lactobacillus rhamnosus* | 0 | 1 | 0 |
| *Lactobacillus sakei* | 1 | 0 | 0 |
| *Paenibacillus polymyxa* | 1 | 0 | 0 |
| *Paeniclostridium sordellii* | 0 | 1 | 1 |
| *Paraclostridium bifermentans* | 1 | 1 | 0 |
| *Phocaeicola vulgatus* | 1 | 1 | 0 |
| *Priestia megaterium* | 0 | 1 | 4 |
| *Raoultella ornithinolytica* | 0 | 1 | 0 |
| *Rothia mucilaginosa* | 1 | 0 | 0 |
| *Staphylococcus carnosus* | 1 | 0 | 0 |
| *Staphylococcus haemolyticus* | 1 | 0 | 0 |
| *Staphylococcus lugdunensis* | 1 | 0 | 0 |
| *Streptococcus agalactiae* | 1 | 1 | 1 |
| *Streptococcus anginosus* | 0 | 2 | 0 |
| *Streptococcus dysgalactiae* | 1 | 0 | 0 |
| *Streptococcus gordonii* | 2 | 1 | 0 |
| *Streptococcus mutans* | 1 | 0 | 0 |
| *Streptococcus parasanguinis* | 0 | 4 | 0 |
| *Streptococcus perosis* | 0 | 1 | 0 |
| *Streptococcus salivarius* | 1 | 4 | 0 |
| *Streptococcus sanguinis* | 0 | 1 | 0 |
| *Streptococcus vestibularis* | 1 | 1 | 0 |
| *Weissella cibaria* | 0 | 2 | 0 |
| *Weissella viridescens* | 0 | 0 | 1 |


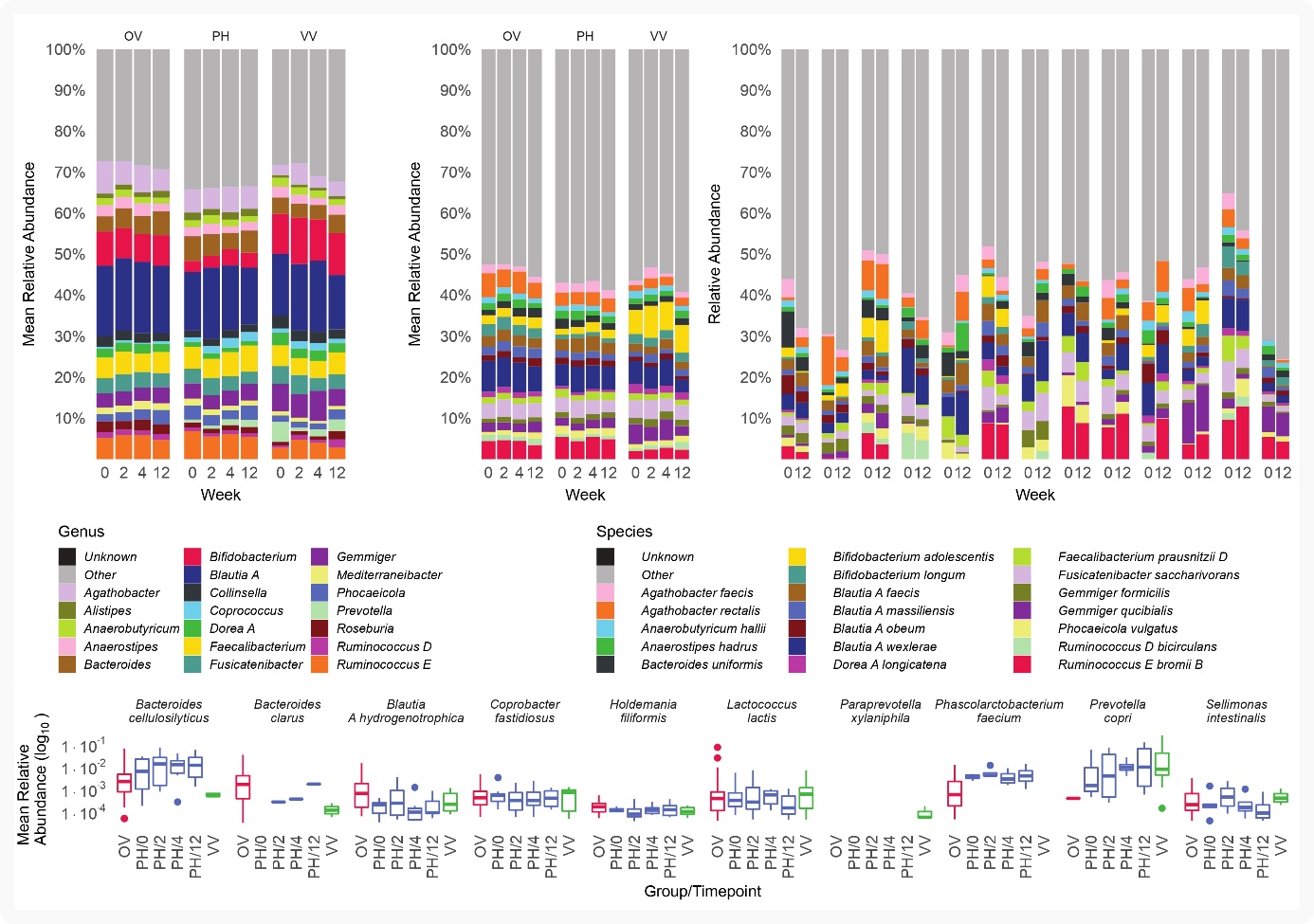


Figure S1: Identical information as displayed in Figure 3 using sourmash for taxonomic profiling of metagenomic reads. Note, the relative amount of unknown taxonomies was removed and information was rescaled. Further, the selected species from Figure 3D were adopted and not recomputed as to highlight abundance differences among workflows.
